# Regulation of inflammatory immune responses leading to the development of bone destructive autoimmune disease rheumatoid arthritis by IL-27

**DOI:** 10.1186/ar3576

**Published:** 2012-02-09

**Authors:** Takayuki Yoshimoto, Mingli Xu, Izuru Mizuguchi, Yukino Chiba, Sadahiro Kamiya, Masanori Matsui, Shiva Shahrara, Junichiro Mizuguchi

**Affiliations:** 1 Department of Immunoregulation, Institute of Medical Science, Tokyo Medical University, Tokyo 160-8402, Japan; 2 Department of Immunology, Tokyo Medical University, Tokyo 160-8402, Japan; 3 Departments of Clinical Sciences, Josai International University, Chiba 283-8555, Japan; 4 Department of Microbiology, Saitama Medical University, Saitama 350-0495, Japan; 5 Department of Medicine, Northwestern University Feinberg School of Medicine, Chicago, IL 60611, USA

## 

IL-27, a member of the IL-6/IL-12 family of cytokines, induces early helper T (Th)1 differentiation and generation of cytotoxic T cells and IL-10-producing type 1 regulatory T cells, while it suppresses the production of inflammatory cytokines and inhibits Th2 and Th17 differentiation [1,2]. The receptor activator of NF-kB ligand (RANKL), which is expressed by not only osteoblasts but also activated T cells, plays an important role in bone-destructive disease rheumatoid arthritis (RA). Recently, IL-17-producing Th17 cells were identified as the exclusive osteoclastogenic T-cell subset. This is because Th17 cells express RANKL, and that IL-17 not only induces RANKL expression on osteoblasts, but also increases the production of various inflammatory molecules. It was previously reported that IL-27 is detected in RA synovial membranes and that treatment with IL-27 attenuated inflammatory responses in collagen-induced arthritis (CIA), one of mouse RA models.

We have been investigating the role of IL-27 in the regulation of inflammatory responses leading to the development of bone destructive autoimmune disease. We first demonstrated that osteoclastogenesis from bone marrow cells induced by soluble RANKL is inhibited by IL-27 with reduced multinucleated cell numbers [3]. Then, other group further clarified that IL-27 directly acts on osteoclast precursor cells and suppresses RANKL-mediated osteoclastogenesis through STAT1-dependent inhibition of c-Fos, leading to amelioration of the inflammatory bone destruction. We recently investigated the mechanistic role of IL-27 in the pathogenesis of CIA and found that local injection of adenoviral IL-27 transcript into the ankles of CIA mice attenuates joint inflammation, synovial lining thickness, bone erosion and leukocyte migration [4]. IL-27 reduced the production of IL-1b and IL-6, and suppressed Th17 cell differentiation as well as IL-17 downstream target genes, which leads to decreased IL-17-mediated monocyte recruitment and angiogenesis possibly through the reduction of neutrophil and monocyte chemokines. We also elucidated that IL-27 inhibits cell surface expression of RANKL on naive CD4^+ ^T cells activated by T cell receptor ligation and secretion of its soluble RANKL as well [5]. The inhibitory effect was mediated in part by STAT3 but not by STAT1 or IL-10. In differentiated Th17 cells, IL-27 much less but significantly inhibited the RANKL expression after re-stimulation.

**Figure 1:**
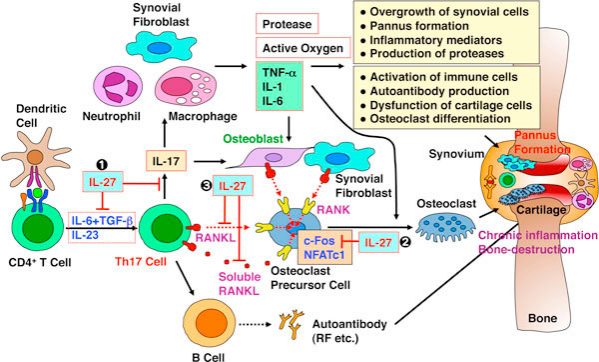
**IL-27 regulates inflammatory immune responses leading to the development of bone destructive autoimmune disease RA through multiple mechanisms**.

Taken together, these results suggest that IL-27 regulates inflammatory immune responses leading to the development of bone destructive autoimmune disease through multiple mechanisms as described above (Figure 1), and that IL-27 may be a promising target for therapeutic intervention to control disease in RA patients.
